# Upregulation of Voltage-Gated Calcium Channel Ca_v_1.3 in Bovine Somatotropes Treated with Ghrelin

**DOI:** 10.1155/2013/527253

**Published:** 2013-12-18

**Authors:** V. M. Salinas Zarate, A. Magdaleno Méndez, B. Domínguez Mancera, A. Rodríguez Andrade, M. Barrientos Morales, P. Cervantes Acosta, A. Hernández Beltrán, D. Romero Salas, J. L. V. Flores Hernández, E. Monjaraz Guzmán, D. R. Félix Grijalva

**Affiliations:** ^1^Laboratory of Cell Biology, School of Veterinary Medicine and Animal Science, University of Veracruz, Veracruz, Mexico; ^2^Department of Chemistry and Biochemistry, Veracruz Institute of Technology, Mexico; ^3^Laboratory of Neuroendocrinology, Institute of Physiology, University of Puebla, Puebla, Mexico; ^4^Department of Cell Biology, Center for Research and Advanced Studies, National Polytechnic Institute, Mexico City, Mexico

## Abstract

Activation of the growth hormone (GH) secretagogue receptor (GHS-R) by synthetic GH releasing peptides (GHRP) or its endogenous ligand (Ghrelin) stimulates GH release. Though much is known about the signal transduction underlying short-term regulation, there is far less information on the mechanisms that produce long-term effects. In the current report, using an enzyme-linked immunosorbent assay for GH detection and whole-cell patch-clamp recordings, we assessed the long-term actions of such regulatory factors on voltage-activated Ca^2+^ currents in bovine somatotropes (BS) separated on a Percoll gradient and detected by immunohistochemistry. After 24 h of treatment with Ghrelin (10 nM) or GHRP-6 (100 nM) enhanced BS secretory activity; GH secretion stimulated by GHS through the activation of GHS-R because treatment with the antagonist of GHS-R (D-Lys3-GHRP-6, 10 *μ*M) blocked the GH secretion, and the effect was dose and time dependent (24, 48, and 72 h). GH secretion stimulated by GHRP-6 was abolished by nifedipine (0.5 *μ*M), a blocker of L-type HVA Ca^2+^ channels, and KN-62 (10 *μ*M), an inhibitor of Ca^2+^/CaM-KII. After 72 h in culture, all recorded BS exhibited two main Ca^2+^ currents: a low voltage-activated (LVA; T-type) and a high voltage-activated (HVA; mostly dihydropyridine-sensitive L-type) current. Interestingly, HVA and LVA channels were differentially upregulated by Ghrelin. Chronic treatment with the GHS induced a significant selective increase on the Ba^2+^ current through HVA Ca^2+^ channels, and caused only a small increase of currents through LVA channels. The stimulatory effect on HVA current density was accompanied by an augment in maximal conductance with no apparent changes in the kinetics and the voltage dependence of the Ca^2+^ currents, suggesting an increase in the number of functional channels in the cell membrane. Lastly, in consistency with the functional data, quantitative real-time RT-PCR revealed transcripts encoding for the Ca_v_1.2 and Ca_v_1.3 pore-forming subunits of L-type channels. The treatment with Ghrelin significantly increased the Ca_v_1.3 subunit expression, suggeting that the chronic stimulation of the GHS receptor with Ghrelin or GHRP-6 increases the number of voltage-gated Ca^2+^ channels at the cell surface of BS.

## 1. Introduction

Growth hormone (GH) synthesis and secretion are regulated by two hypothalamic hormones with opposite actions, the GH releasing hormone (GHRH) and somatotropin release inhibiting factor (SRIF; somatostatin) [1]. However, the availability of GH secretagogues (GHS) like Ghrelin [2] and its synthetic homologue, the GHRP-6, have increased the interest on how these GHS participate in GH release.

It has been demonstrated that, in addition to neurons and muscular cells, endocrine cells like somatotropes, express spontaneous electrical activity; which means that they are electrically excitable and able to produce action potentials (AP) by activation of diverse populations of voltage-sensitive ionic channels. It is well established that voltage-gated ion channels are involved in the control of the excitation-secretion coupling that leads to hormone release in different pituitary hormone-secreting cell types, including somatotropes [3]. Of these ionic channels, the low and high voltage activated Ca^2+^channels (Ca_v_ LVA-T and HVA-L-type) and Na^+^ tetrodotoxin-sensitive (Na_v_ TTX) channels play a role in the rising phase of the AP (depolarization) in the cell membrane whose result is the increase of the [Ca^2+^]_*i*_ and the GH release. Diverse intracellular signaling systems are suggested to mediate the acute actions of the GHS. After binding of ligand, GHS-R acts through the G*α*_11_ subunit of G-protein to activate phospholipase C (PLC) resulting in hydrolysis of PIP_2_ to generate inositol 1,4,5-triphosphate (IP_3_) and diacylglycerol (DAG) [4]. As a consequence, [Ca^2+^]_*i*_ increases due to a transient release of Ca^2+^ from IP_3_-responsive cytoplasmic storage pools and a sustained Ca^2+^ influx due to activation of voltage-activated Ca^2+^ channels and blockade of K^+^ channels [5], resulting in a depolarization of the somatotrope membrane and release of GH [4, 6]. It is known that gonadotropes cells exhibit spontaneous low-amplitude fluctuations in [Ca^2+^]_*i*_ due to intermittent firing of nifedipine-sensitive action potentials. Membrane potential (*V*_*m*_) and Ca^2+^ oscillations are interdependent during continued gonadotropin-releasing hormone action, and Ca^2+^ entry is necessary for the maintenance of [Ca^2+^]_*i*_ spinking. The initial and sustained Ca^2+^ transients induced by the agonist and *V*_*m*_ oscillations are abolished by blockade of endoplasmic reticulum Ca^2+^-ATPase, consistent with the role of Ca^2+^ reuptake by internal stores in the oscillatory response during both phases. Such a pattern of synchronization of electrical activity and Ca^2+^ spiking in cells regulated by Ca^2+^-mobilizing receptors shows that the operation of the cytoplasmic oscillator can be integrated with a plasma membrane oscillator to provide a long-lasting signal during sustained agonist stimulation [7]. Voltage-gated Ca^2+^ influx plays important roles during sustained receptor activation. The effects of these receptors on calcium channel expression could be of relevance for GHRH action, which exclusively operates through cAMP signaling pathway and promotion of voltage-gated calcium influx [1].

In GC cells (a somatotrope tumor cell line) treated by GHRP-6 it has been observed that GHS increases GH release activating a G-protein coupled to the GHS receptor (GHS-R), which activates the IP_3_-PKC signaling pathway [8]. In the same way, the upregulation of voltage-gated HVA L-type calcium channel (Ca_v_1.3) [9] and an upregulation of voltage-gated Na^+^ channels by long-term activation of the GHS-R in this cell line have been observed [8]. Likewise, in goldfish pituitary cells Ghrelin-induced GH release involves voltage-sensitive calcium channels [10, 11]. Moreover, Ghrelin inhibits proliferation and increases T-type Ca^2+^ channel expression in PC-3 human prostate carcinoma cells [12]. These effects were analyzed using goldfish and tumoral cells, but the same effects could be found in normal cells and other species like mammals. In bovines, the negative energetic balance induces an increase in the circulating Ghrelin concentration [13] that is produced by abomasum principally [14]; this effect is counteracted after feeding [15]. Rat Ghrelin (rGhr) has been tested in bovine cultured somatotropes, resulting in a higher releasing response than in cells treated with GHRH and human Ghrelin [16]. In in vivo ruminants, it has been observed that inoculation of rat Ghrelin (rGhr) increases immediately (15 min) the bovine GH (bGH) blood concentration and it has been registered that bGH release increase is dose dependent [17].

In spite of this active research, the cellular mechanism implicated in the bGH releasing effect of GHS has not been fully described; therefore, in the current report, we examined whether chronic treatment with Ghrelin or GHRP-6 affects the functional expression of plasma membrane voltage-activated Ca^2+^ channels. Our results suggest an upregulation in the functional expression of HVA (Ca_v_1.3) Ca^2+^ channels by GHS when chronically applied to bovine somatotropes and that this effect is compatible with an increase in hormone secretion.

## 2. Materials and Methods

### 2.1. Chemicals

Ghrelin (ALX-157-022-M001), tetrodotoxin (BML-NA120-0001), KN-62 (BML-EI230), and nifedipine (ALX-550-091-G005) were purchased from Enzo-Alexis-Biomol (ENZO Life Sciences), GHRP-6 (HOR-298-1) was purchased from ProSpec protein specialist, and D-Lys3-GHRP-6 (G-4535-5MG) was purchased from Sigma (St. Louis, MO). All other chemicals were of reagent grade.

### 2.2. Glands Sampling

Heads from male bovine predominantly European breeds were obtained from a local slaughterhouse; bovines were sacrificed by humanitarian methods according to the Mexican norm NOM-033-ZOO-1995. Pituitary glands were surgically taken out during the first hour *postmortem*. A histological analysis, staining with haematoxylin-eosin stain [18], was performed to verify the correct extraction of the gland.

### 2.3. Somatotrope Enriched Population

Pituitary glands were dissociated as previously described [19]. Once removed, the pituitary gland the anterior lobe was cut to 2-3 mm cubes by a scalpel; after this, enzyme disaggregation was done using papain (3 mg/mL) at 37°C in a trypsinization flask with a magnetic stirrer for 45 min until complete disaggregation occurred. Cells were collected and washed in PBS (Phosphate Buffer Solution, Sigma) after each cycle. Percoll suspension was prepared by diluting the 100% Percoll (Sigma) with the appropriate proportions of 10x concentrated in PBS, at percentages 10, 20, 30, 40, and 50% (densities 1.036, 1.048, 1.059, 1.071, and 1.083 g/cm^3^, resp.) [20], for all cell separation. In a 15 mL centrifuge tube (SARSTEDT), the gradient was built up using 2 mL of each density suspension. The cell suspension was layered on top of the gradient and centrifuged for 35 min at 830 g.

For detection of somatotropes, an immunocytochemical stain (Millipore), using a specific polyclonal bGH antibody (Rb X Growth Hormone, Millipore AB940) was evaluated in each Percoll density. The cells in fraction 40% showed to have a higher somatotropes proportion (>80%) and with a viability >90%; they were planted in petri dishes of 35 mm 6-well plates or flasks (25 cm^2^) (SARSTEDT), depending on the experiment to be performed. Viability was determined by the Trypan-blue exclusion method using a hemocytometer [19].

### 2.4. Cell Culture

Cultures were maintained inside a humid CO_2_ incubator at 37°C during experimental time. Cells were grown with Dulbeceo's Modified Eagle Medium (DMEM High Glucose, Biowest), supplemented with 10% fetal bovine serum (Biowest), 100 IU/mL penicillin and 100 *μ*g/mL streptomycin (Sigma), and 5% of L-glutamine (Sigma).

### 2.5. Electrophysiology

Ca^2+^ current recordings were performed in bovine somatotropes under control conditions or after chronic treatment with Ghrelin (10 nM) or GHRP-6 (100 nM), according to the whole-cell mode of the patch-clamp technique [22], using an Axopatch 200B patch-clamp amplifier (Molecular Devices, Foster City, CA) and acquired on-line using a Digidata 1440A interface with pClamp10 software (Molecular Devices). After establishing the whole-cell mode, capacitive transients were cancelled with the amplifier. Leak and residual capacitance currents were subtracted online by a P/4 protocol. Current signals were filtered at 5 kHz (internal 4-pole Bessel filter) and digitized at 10–100 kHz. The bath recording solution contained (in mM) 133 NaCl, 10 TEA-Cl, 10 BaCl_2_, 10 HEPES, 5 glucose, and 0.001 TTX (pH 7.3 NaOH). The internal solution consisted of 100 CsCl, 30 NaCl, 2 MgCl2, 1 CaCl2, 10 EGTA, 10 HEPES, 2 Na-ATP, 0.05 GTP, and 5 glucose (pH 7.3 CsOH). Experiments were performed at room temperature (22°C). Control and treated cells were rinsed with peptide-free culture medium and maintained in this medium for ~60 min before membrane currents were recorded. Membrane capacitance (Cm) was determined as described by Avila et al. [23] and used to normalize currents. Briefly, Cm values were determined by applying a series of three consecutive pulses (10 ms; −10 mV) from a holding potential (−80 mV) and integrating the current traces that resulted from subtracting the capacitive transients associated with the patch pipette (cell-attached conditions) from the total capacitive transients obtained immediately after breaking into the cell (whole-cell conditions).

### 2.6. Enzyme-Linked Immunosorbent Assay for GH Detection

Release of GH from bovine somatotropes treated with Ghrelin or GHRP-6 was quantified using a commercial enzyme-linked immunosorbent assay (ELISA) kit. The medium samples were collected after the treatments time and stored at −21°C until the analysis. The color intensity of the reaction product (proportional to the concentration of bGH) was measured by spectrophotometry in a plate reader using a 450 nm filter (BIO RAD Model 680). Intensity values of the samples were compared with values in a standard curve using Sigma Plot 11.0 software (Systat Software, Chicago, IL). Standard curve was made using bovine somatotropin following the kit protocol (Uscnk Life Science, Inc., Wuhan; enzyme-linked immunosorbent assay kit, for bovine growth hormone; catalog number E90044Bo). The amount of bGH (pg) dissolved in the medium was expressed in proportion for each 10 mg of total protein (bGH pg/10 mg TP), which was determined by the Bradford method [24]. Briefly, the technique consists in recovering and lysing the cells and centrifuge them at 12,000 g for one minute; 2 *μ*L of sample diluted in 98 *μ*L of deionized water is mixed with 100 *μ*L of Coomassie-Blue (G-250) mix solution and measured by spectrophotometry (Spectronic Genesys 5) at 595 nm. A standard curve was made to analyze protein concentrations using bovine serum albumin (BSA 1 mg/mL, Sigma) as standard protein.

### 2.7. Ca_v_ mRNA Expression

Enriched somatotrope populations were treated with GHS for 3 h simultaneously with their respective control group. The mRNA was isolated with the Zymo Research, Quick-RNA MiniPrep Kit (Catalog R1054). Briefly, the method consists in recovering and lysing the cells with the ZR RNA buffer; the lysate is transferred into a column in a collection tube and centrifuged at 12,000 g for 1 min. Total RNA was purified with RNA prewash buffer, RNA wash buffer, and DNase/RNase-free water. Eluted RNA was stored at ≤20°C until its analysis. RNA quality was measured by spectroscopy (BioPhotometer Eppendorf), and samples were accepted when integrity A260 nm/A280 nm ≥1.6 and RNA concentration ≥3 *μ*g/10 *μ*L were reached.

A coupled retrotranscription was performed with the commercial kit High Capacity cDNA Reverse Transcription (Applied Biosystem) following the kit protocol. Primers (Table 1) for each ionic channel studied, bGH gene, and *β*-actin were designed for measuring gene expression following the kit Pyrostart fast PCR Master Mix (Fermentas) procedure with the PCR protocol 94°C/5 min, 35 cycles 94°C/30 sec + 57.5°C/30 sec + 72°C/1 min, and 72°C/10 min.

The amplified PCR products visualization was resolved by electrophoresis in 1.2% agarose gels and stained with ethidium bromide for 65 V/90 min; TAE was used as running buffer and the marker Gene Ruler 100 pb DNA ladder was also used (Biosis). The bGH was run to demonstrate the presence of somatotropes and that *β*-actin was used as endogenous control for gene expression. Semiquatitative analysis was performed with the software ImageJ.

### 2.8. Statistical Analysis

Data were analyzed and plotted by the combined use of pCLAMP software and Sigma Plot software (SPSS, Chicago, IL) and are given as mean ± SE. Statistical differences between two means were determined by Student's *t*-tests (*P* < 0.05). Curve fits were made using the nonlinear, least-squares fitting procedure included in the Sigma Plot program. The bGH mean concentration mean ± SE for each treatment was expressed as percentage and compared quantitatively with the control. One-way ANOVA analysis was performed for bGH release between the distinct treatments, and for Ca_v_ mRNA expression and quantitation using the Tukey method for mean comparisons (*P* < 0.05).

## 3. Results

**Figure 1:**
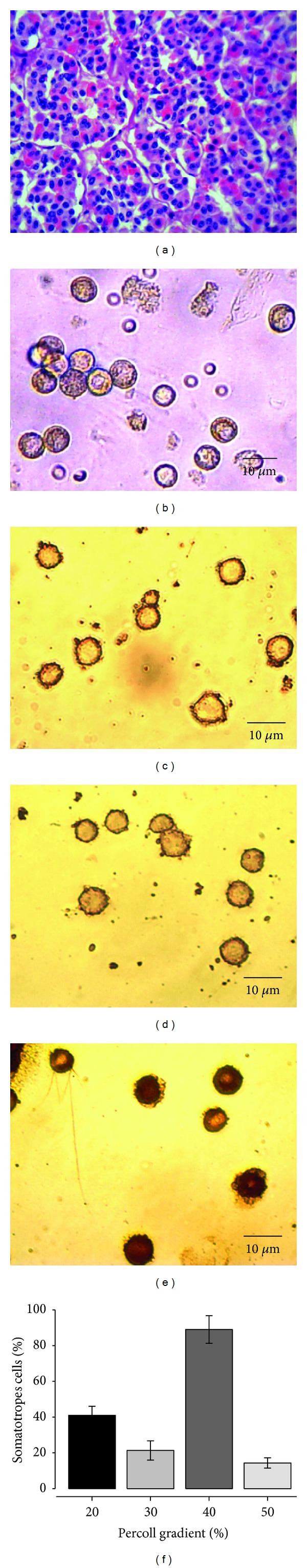
Bovine somatotropes. (a) Histological image (20x) of bovine pituitary stained with haematoxylin-eosin stain. (b) Bovine pituitary cells in primary culture. ((c), (d), and (e)) Bovine somatotropes ICC stained (c) in the absence of specific bGH primary antibody, (d) exposed to the specific bGH primary antibody without the second antibody, and (e) exposed to both specific bGH primary antibody and secondary antibody. (f) Percentage of bGH positive cells found in each Percoll gradient fraction.

Bovine pituitary glands (Figure 1(a)) were dispersed and cultured in DMEM (see Materials and Methods); and the cells maintained in primary culture up to seven days (Figure 1(b)). In order to separate somatotrope cells, (GH cells) a four-layer discontinuous density gradient was used, and GH cells were immonocytochemically stained (ICC) for determining the proportion of GH cells (Figures 1(c), 1(d), and 1(e)). The 1.071 (40%) fraction showed the higher number of immunoreactive cells (Figure 1(f)).

**Figure 2:**
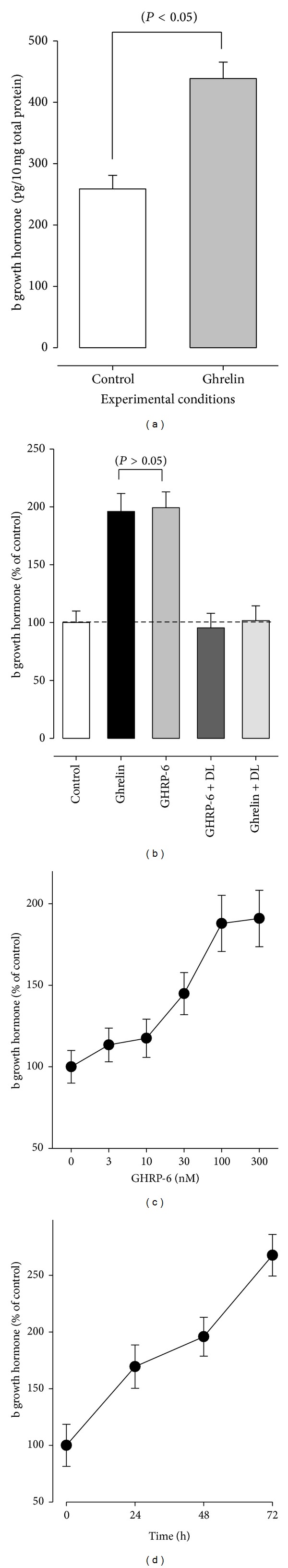
Ghrelin and GHRP-6 enhance secretory activity in bovine somatotropes. (a) Ghrelin (10 nM) significantly stimulates GH secretion in bovine GH cells. (b) GH-stimulated secretion by GHS is mediate through GHS-R activation because the treatment with the antagonist of GHS-R (DL3-GHRP-6) blocked GH secretion; Ghrelin (10 nM), GHRP-6 (100 nM), GHRP-6 (100 nM) + DL3-GHRP-6 (10 *μ*M), Ghrelin (10 nM) + DL3-GHRP-6 (10 *μ*M). (c) Dose response curve of GH secretion stimulated by GHRP-6 (48 h). (d) Time course of GH secretion stimulated by GHRP-6 (100 nM).

Isolated somatotropes were then treated for 24, 48 and 72 h with Ghrelin (10 nM) or GHRP-6 (100 nM), and the results indicate that GHS specifically stimulate GH secretion (Figure 2(a)). When the antagonist of GHS-R, DL-3 GHRP-6 (10 *μ*M) the GH secretion stimulated by GHS was blocked (Figure 2(b)) showing that GH secretion simulated by the GHS is mediated through the activation of GHS-R and that this effect is dose (Figure 2(c)) and time dependent (Figure 2(d)) on GHRP-6.

**Figure 3:**
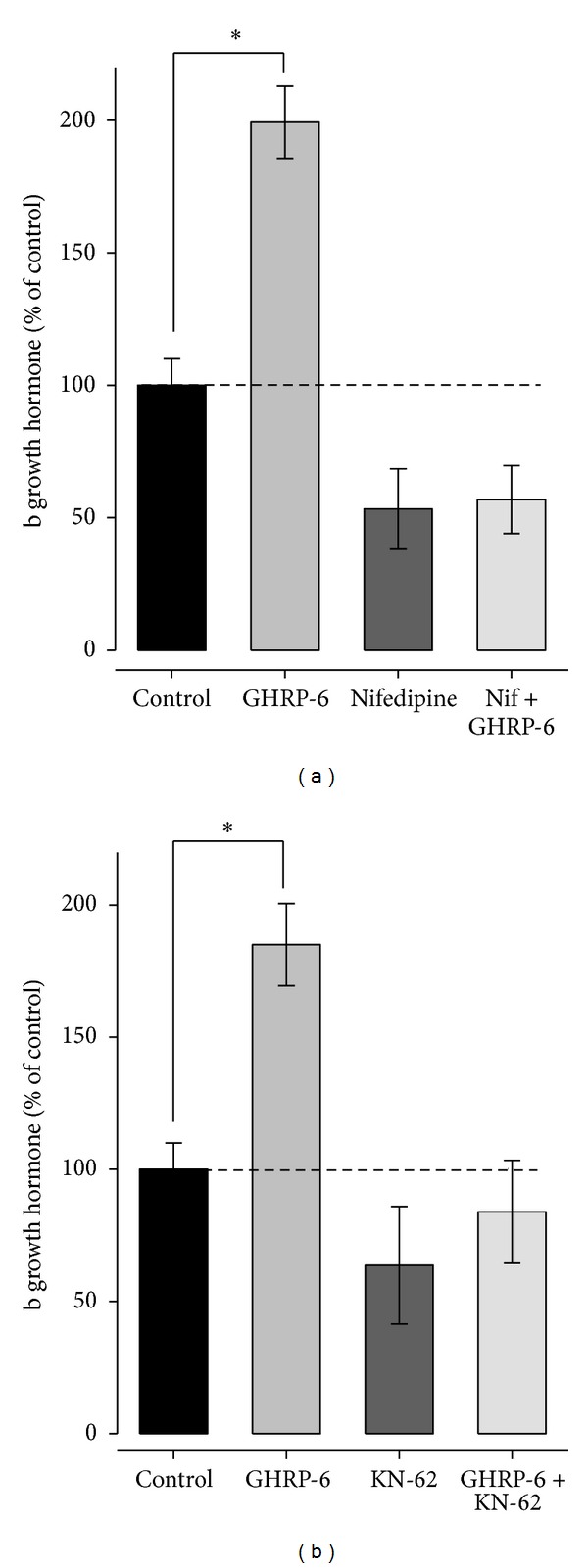
High voltage activation calcium channels' L-type regulation and pathway signaling Ca^2+^/CaM-K II affect GH release in bovine somatotropes. (a) Bar graph illustrating the regulation of hormone release by GHRP-6 (100 nM) applied alone or in the presence of nifedipine (0.5 *μ*M) a blocker of HVACC L-type. (b) Average amount of GH released in the absences (control) and after treatment with the signaling pathway Ca^2+^/CaM-K II inhibitor KN-62 (10 *μ*M) alone or in combination with GHRP-6 (100 nM). Each value represents the mean ± SE of determinations performed in triplicate from three independent experiments. The asterisks denote significant differences (*P* < 0.05) as compared with the control untreated cells.

In other series of experiments, bGH secretion was measured through the activation of high voltage activation L-type calcium channels (HVACC) and the activation of the Ca^2+^/CaM-K II siganaling pathway. To this end, nifedipine (0.5 *μ*M) was used to block L-type channels and KN-62 (10 *μ*M), to block the Ca^2+^/CaM-K II siganaling pathway. Our results indicate that bGH secretion induced by GHRP-6 is mediated by L-type Ca^2+^ channels, when using nifedipine, the stimulatory effect of GHRP-6 is not shown (Figure 3(a)); in the same way, KN-62 blocked the GH secretion stimulated by GHRP-6, revealing that GHS promote the GH secretion promoting calcium entry from the extracellular medium by activating the pathway signaling Ca^2+^/CaM-K II.

**Figure 4:**
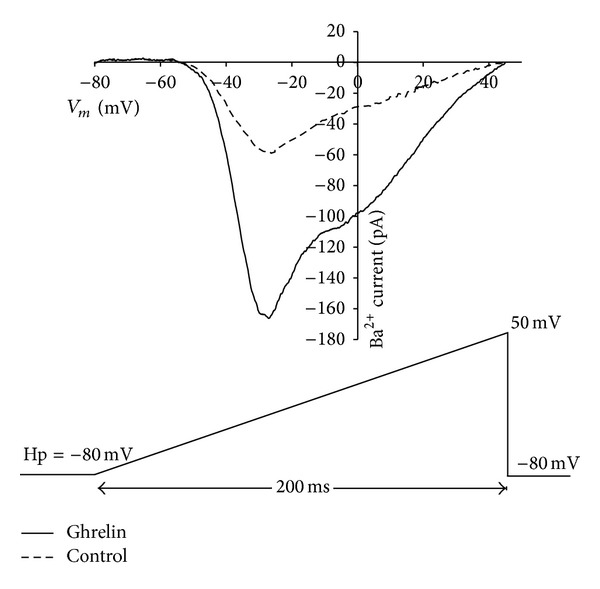
Calcium channel activity in bovine somatotropes. A ramp protocol from a holding potential of −80 mV to 50 mV with a duration of 200 ms was used to evocate the tow components of the current. Short dash line, control current trace; Solid line, 48 h treatment with Ghrelin (10 nM).

**Figure 5:**
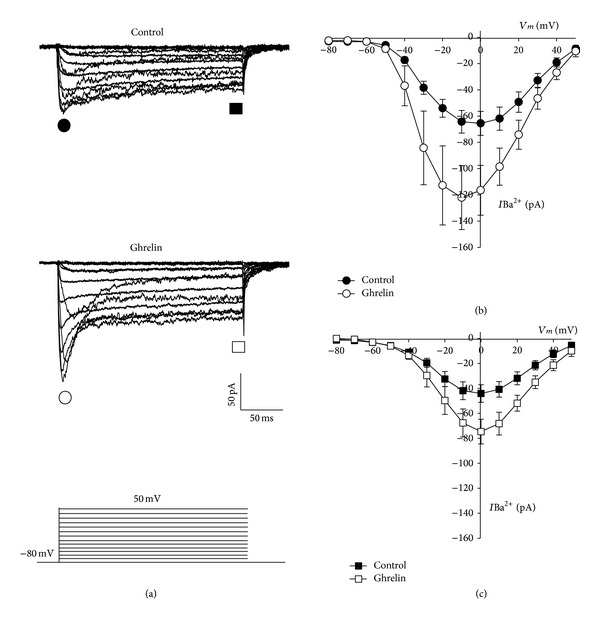
Treatment with Ghrelin enhances Ca^2+^ channel activity in bovine somatotropes. (a) Voltage-activated Ba^2+^ currents (through Ca^2+^ channels) evoked by depolarizing test pulses from a Vh of −80 mV with a 10 mV interval (as indicated in the lower panel) in bovine GH cells kept in culture in the absence (control) or presence of Ghrelin (10 nM) for 48 h. (b) Average current-voltage (*I*-*V*) relationships obtained from control (*⬤*, *n* = 5) and treated cells (*⚪*, *n* = 6) measured at the peak of each current trace. (c) Average *I*-*V* curves obtained as in (a); current values were obtained at the end of the command pulse (□: Control; ■: Ghrelin).

In order to assess whether the chronic treatment with GHS is associated with a modification of voltage-activated Ca^2+^ channel activity, we initially characterized the macroscopic calcium currents using Ba^2+^ as a charge carrier in bovine somatotropes. Whole-cell patch-clamp recordings confirmed the expression of two types of Ca^2+^ currents in the plasma membrane of these cells. When depolarization was carried out from a holding potential (*V*_*h*_) of −80 mV in a ramp protocol, the two components were observed in both, control and treated cells with Ghrelin (10 nM) (Figure 4). The first component activated at potential more negative than −40 mV and the other component can be seen at potentials > −10 mV; when the ramp protocols were changed for 10 mV steps, the two components were seen more clear (Figure 5(a)). A rapidly activating inward current that inactivated within 50 ms of pulse onset and a second, higher-threshold current component were also observed, which were activated at around −40 mV and only partially inactivated during a 200 ms pulse. In GH cells treated with Ghrelin for 48 h, a current increase was observed in the two components, peak and sustained (Figures 5(b) and 5(c)).

**Figure 6:**
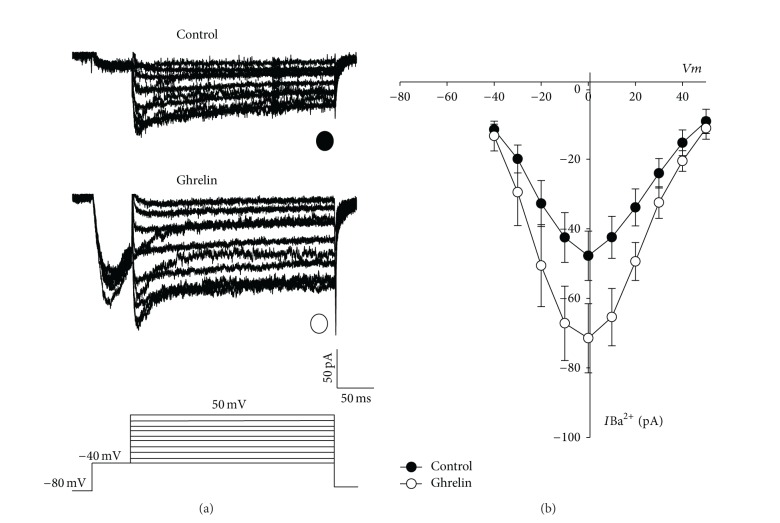
Calcium channel activity in bovine somatotropes. (a) Family of current traces obtained from a control and treated cell with Ghrelin (10 nM) for 48 h; the holding potential is fixed to −80mv, and after that a prepulse was clamped at −40 mv with a duration of 50 ms was applied to inactivate the low voltage-gated calcium channels. (b) Average current-voltage (*I*-*V*) relationships obtained from control (*⬤*, *n* = 5) and treated cells (*⚪*, *n* = 6) measured at the end of the command pulse.

When depolarization was carried out from a prepulse to −40 mV with a *V*_*h*_ of −80 mV to inactivating the low voltage gated calcium channels (Figure 6(a)), the amplitude of *I*Ba^2+^ was decreased in all cells under study. Current amplitude was measured at the end of the pulse (195 ms) and the *I*-*V* relationship is shown in Figure 6(b). A bell-shaped dependence on the command pulse potential with a threshold at −40 mV and a maximum at depolarization to 0 mV was found in all cells analyzed, and the treatment with Ghrelin for 48 h increased current at each depolarization step.

**Figure 7:**
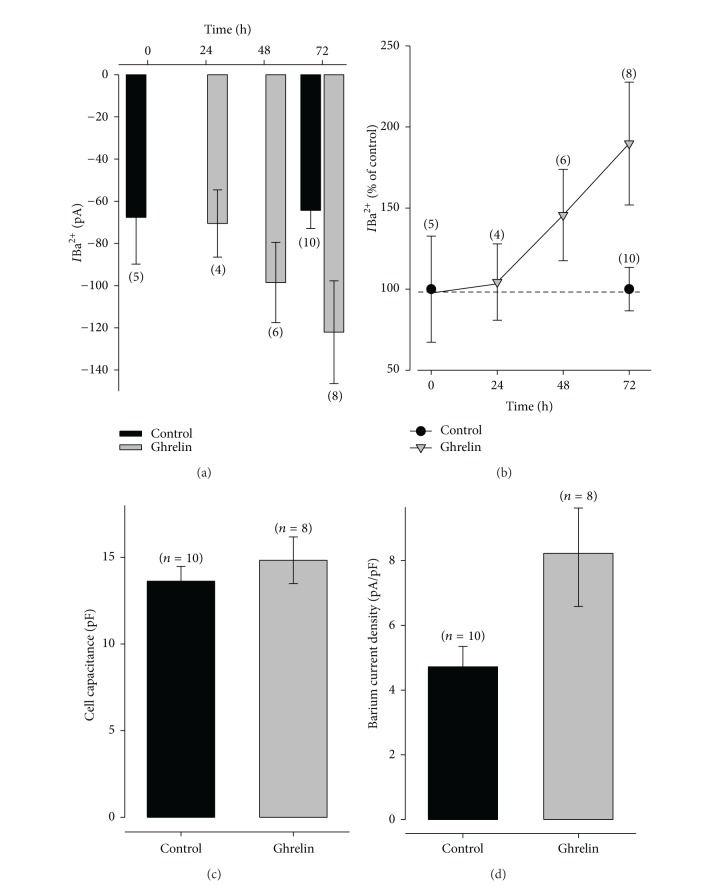
Exposition time dependence to Ghrelin (10 nM) of the barium current in bovine somatotropes. (a) Average of the barium current measured at peak (50 ms) at different times treatment with Ghrelin (10 nM), the number in parentheses indicate the mumber of cells analyzed. (b) Comparison of current percentage at different times after Ghrelin treatment data were taken from the part (a). (c) Cell capacitance of somatotropes at 72 h of treatment and in control condition. (d) Current density from bovine somatotropes treated with Ghrelin 10 nM and control cells.

In this series of experiments, we investigated if *I*_Ba_ through Ca^2+^ channels shows a dependence on the time treatment. To this end, BS were treated for 24, 48, and 72 h and the results are shown in Figure 7. Our results indicate that at least 24 h of exposition with Ghrelin (10 nM) is necessary to show an increment in current (Figure 7(a)), and that the maximal effect is seen at 72 h (2-fold increase) (Figure 7(b)), without affecting cell capacitance (Figure 7(c)). In addition to eliminate the cell size as a variation source we estimated current density (Figure 7(d)) and the results show that Ghrelin treatment increases current density in bovine somatotropes cells.

**Figure 8:**
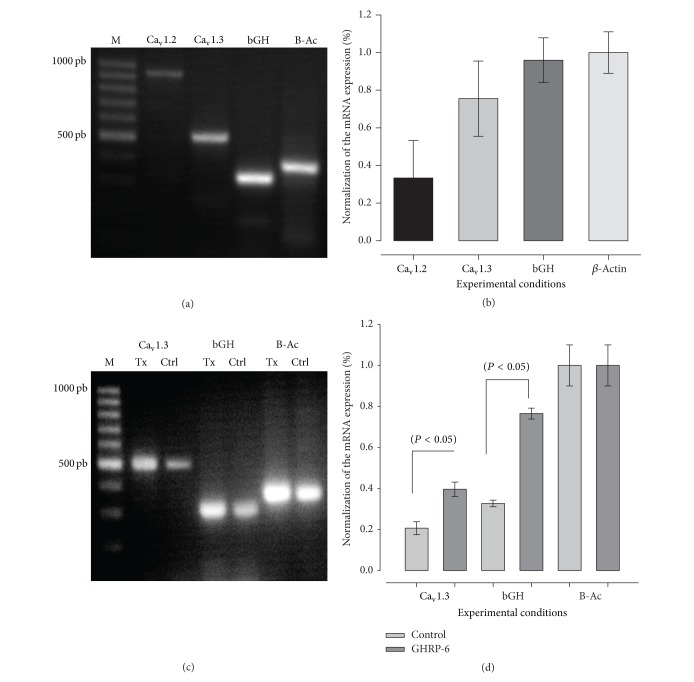
Expression of L-type Ca^2+^ channel *α*1 subunits mRNA in bovine somatotropes. (a) Electrophoresis of L-type Ca^2+^ channel a 1.2 and 1.3 subunits mRNA, bGH, and *β*-actin (B-Ac). (b) Summary of three independent experiments; the principal subunit expressed is 1.3. (c) Electrophoresis of somatotropes cells treated by GHRP-6 100 nM for 48 h. (d) Summary of three independent experiments; GHRP-6 increases the mRNA for HVA calcium channels L-type 1.3 and the mRNA for bGH.

Last, to evaluate if the secretagogues have an effect on the mRNA encoding for the principal subunit Ca_v_1.2 (*α*1C) and Ca_v_1.3 (*α*1D) of L-type calcium channels and bGH, a group of experiments were carries out Somtotropes were treated with GHRP-6 (100 nM) for 48 h and Figure 8(a) shows that the principal subunit of L-type channels expressed in bovine somatotropes was Ca_v_1.3. A summary of these results is showed in Figure 8(b); treatment with GHRP-6 increases ~2-fold the mRNA encoding this protein; in addition, the mRNA that encodes bGH was increased ~2.1-folds. Therefore, the increase of functional HVA channels in bovine somatotropes may be mediated by enhanced transcription of the ion-conducting subunit of L-type Ca^2+^ channel subunits particularly Ca_v_1.3.

## 4. Discussion

There are two levels in the control of GH secretion: the exocytotic pathway responsible for release of prestored hormone (short term) and *de novo* synthesis (long term). Though much is known about the regulation of GH secretion in the short term by GHRH and Ghrelin or its synthetic analog GHRP-6, little is known about the cellular mechanisms exerted by these secretagogues on GH secretion, such as signaling pathways, molecular mechanisms, and expression of specific genes.

Growth factors, hormones, and neurotransmitters have both short-term and long-term regulatory effects on the activity of HVA voltage-activated Ca^2+^ channels. Though much is known about the signal transduction underlying short-term regulation, but there is far less information on mechanisms that produce long-term effects. Although the molecular mechanisms of GH release are ill defined, some studies have shown that GHS-R sustained activation may be linked to increased voltage-gated Ca^2+^ channel surface expression, resulting in enhanced electrical and secretory activity in clonal pituitary somatotropes [9, 25]. This latter type of regulation is likely to play a role in long-lasting forms of plasticity in the pituitary gland [26]. Hence, it has been reported that chronic treatment with agonists of the dopaminergic D_2_ receptors downregulates HVA Ca^2+^ channel expression, leading to a reduction in Ca^2+^ current and secretory activity in cultured melanotropes [27, 28]. On the other hand, chronic treatment with epidermal growth factor has been shown either to increase [29] or to decrease [30], HVA Ca^2+^ current density, while nerve growth factor [31] or glucocorticoid hormones [32, 33] significantly enhance HVA current density in clonal pituitary cells.

Though the molecular basis of these regulatory actions has not been yet determined, it has been suggested that they possibly result from alterations in gene expression that ultimately produce changes in the number of functional channels in the plasma membrane. For example, chronic activation of D_2_ receptors results in decreased cAMP levels and Ca^2+^ influx that down-regulate the expression of the messenger ribonucleic acid (mRNA) encoding the pore-forming *α*1D subunit of the L-type Ca^2+^ channels [28], while dexamethasone and natural glucocorticoids significantly increase *α*1C mRNA expression [33]. Thus, hormones and neurotransmitters may produce long-term effects on Ca^2+^ homeostasis in pituitary cells by differentially regulating expression of Ca^2+^ channel subunit genes.

It is worth mentioning that cell heterogeneity in the pituitary has always complicated the study of long-term Ca^2+^ channel regulation in GH-secreting cells. However, a long-term effect of GHS on voltage-activated inward currents has been reported in the relatively simple system of the GC cells [8, 9, 25], which represents a homogeneous *in vitro* model of tumor somatotropes [34, 35]. Consistent with this, the results shown in the present report strongly suggest that current density through HVA Ca^2+^ channels can undergo marked changes in response to the chronic influence of GH secretagogues in bovine somatotropes. In particular, we found that whole-cell conductance through fast deactivating (HVA) Ca^2+^ channels was significantly increased after long-term exposure to GHS. This stimulation of channel activity was prevented by chronic treatment with a specific antagonist of the GHS-R, the D-Lys3-GHRP-6, measured trough GH secretion. On the other hand, slow deactivating (LVA, low voltage activation) channels apparently showed an increase in current amplitude, but more experiments are needed to be conducted for clarify this effect.

The voltage dependence of the currents did not change significantly with GHS treatment; we speculate that the increase in current amplitude in treated BS might preferentially be due to the increase in the surface channel density. Apparently, the L-type is the most prominent Ca^2+^ current component in the bovine somatotropes, and the depolarizing spiking phase of action potentials in GH-secreting cells is probably mediated by this type of channels [35–37]. GHS stimulate GH release in many species like swine [38], bovine [17], and goldfish pituitary cells that involve voltage-sensitive calcium channels [10], but this effect has been only analyzed under acute treatment.

Likewise, several of the genes encoding the ion-conducting subunits of the HVA channels have been found to be expressed in GH-secreting cells [21, 39, 40]. Though the role of each channel subtype in these cells remains unclear, most studies conclude that L-type calcium channels are primarily involved in GH secretion. In this study, we first wanted to determine the level of expression of L-type Ca^2+^ channel genes. The genes that we tested were the Ca_v_1.2 and Ca_v_1.3. We found that mRNA for both channel subunits was present in bovine somatotropes. The quantitative analysis of mRNA revealed that the Ca_v_1.2 is less expressed in the control condition; after the treatment, our data suggest that Ca_v_1.3 mRNA is expressed at a higher level when cells are exposed to the GH secretagogue.

Testing whether Ghrelin and GHRP-6 may differentially stimulate expression of distinct HVA or LVA Ca^2+^ channel subunit genes is an interesting topic for future experiments. However, the possibility that the long-term stimulation of the GHS-R somehow changed the activity of other factors that activate transcription of Ca^2+^ channels is even more challenging. Likewise, GHS-induced increase in L-type Ca^2+^ channel activity could occur either by direct gene regulation or by posttranslational processing.

Interestingly, it has been reported that acute treatment with Ghrelin increases [Ca^2+^]_*i*_ [38] and GH release [41] in somatotropes by a mechanism that may involve L-type Ca^2+^ channel activation. In a larger time scale, this may provide an effective mechanism for up-regulation of electrical activity and voltage-activated Ca^2+^ influx. Ca^2+^ entry through HVA channels might increase hormone synthesis by promoting GH gene expression. Therefore, the stimulatory action of GHS on HVA Ca^2+^ current density would be an increased synthesis and release of GH associated to a larger entry of Ca^2+^ during SAP firing. This may explain, at least in part, the clinical effectiveness of GHS in enhancing GH release [4, 42]. Based on these results, we speculate that long-term exposure to GHS might increase Ca^2+^ influx in bovine somatotropes and could stimulate the expression of (a) transcription factor(s) that promote(s) Ca^2+^ channel synthesis. In this regard, it is worth noting that nuclear Ca^2+^ is an important regulator of gene expression following membrane depolarization of excitable cells. Hence, nuclear Ca^2+^ transients in neurons activate gene transcription by a mechanism that involves the cAMP response element (CRE) and the CRE-binding protein, CREB [43]. In hippocampal neurons, for example, Ca^2+^ influx through L-type channels (and N-methyl-D-aspartate receptors) is capable of causing rapid translocation of Ca^2+^/CaM-K II/IV to the nucleus, which is important for CREB phosphorylation [44]. Signaling pathways mediating the major neuroendocrine regulators of mammalian somatotropes reported by Chang et al. [45] include membrane voltage-sensitive ion channels, Na^+^/H^+^ antiport, Ca^2+^ signaling, multiple pharmacologically distinct intracellular Ca^2+^ stores, cAMP/PKA, PKC, nitric oxide, cGMP, MEK/ERK, and PI3K.

The actions of GHS treatment on pituitary somatotropes are linked to G-protein-coupled GHS-R [6], and GHS-stimulated GH release depends on the cAMP/PKA and PLC/PKC systems and extracellular Ca^2+^ influx [41, 46, 47]. Likewise, it is well known that various kinases are capable of phosphorylating CREB, including Ca^2+^/CaM-K II/IV [48, 49]; another finding of our work is the fact that the inhibitor of Ca^2+^/CaM-K II, KN-62 abolished the GHS-induced GH secretion in bovine somatotropes. These results suggest that Ca^2+^/CaM-K II plays a role in the signaling pathway from GHS-R activation to an increase in Ca^2+^ channel functional expression. Work by other groups it has shown that using isoquinolinesulfonamides KN-62 and KN-93 (calmodulin-dependent protein kinase inhibitors) and KN-92 (an inactive analog) blocked basal prolactin release in a dose- and time-dependent manner, suggesting that calmodulin-dependent protein kinase could mediate the coupling of electrical activity and secretion. However, a similar effect on basal prolactin release was observed on application of KN-92, which does not inhibit this kinase; and therefore caution should be taken when interpreting data from studies using isoquinolinesulfonamides to evaluate the role of calmodulin-dependent protein kinases in excitable endocrine cells, because inactive compounds exhibit comparable effects on action potential secretion coupling to those of active compounds [50]. Additional work on the promoter region of the Ca^2+^ channels expressed in bovine somatotropes will be needed to define the role of nuclear Ca^2+^ and Ca^2+^/CaM-K II in intracellular protein cascades leading to activation of gene transcription after GHS treatment.

In conclusion this study demonstrates that the chronic stimulation of the GHS receptor with Ghrelin or GHRP-6 increases voltage-gated Ca^2+^ channels at the cell surface of bovine somatotropes.

## Figures and Tables

**Table 1:** Primers sequences used in the PCR reactions [21].

Locus	Sequence (5′-3′)	bp.	Name	GenBank
Ca_v_1.2 (*α*1c)	FW 5′-CGAAGCTTCTTCATGATGAACATCT-3′	928	Q704P5	AJ621048.1
	RV 5′-GCGGATCCATGTAGAAGCTGATGAA-3′			
Ca_v_1.3 (*α*1D)	FW 5′-GATCTGGCAAAACAGTGATTTC-3′	510	Q704P3	AJ621050.1
	RV 5′-GTGAAGACCATGTTCAGAATGT-3′			
*β*-Actin	FW 5′-GGCCCAGAGCAAGAGAGGCA-3′	368	ACTB	NM_173979.3
	RV 5′-GGTCCAGACGCAGGATGGCA-3′			
bGH	FW 5′-TGTGGACAGCTCACCAGCTATGAT-3′	314	BTGHG1	AF117346.1
	RV 5′-GTCTGATTTCTGCTGGGCCTCAT-3′			
